# Quali-Quantitative Analysis (QQA): Why It Could Open New Frontiers for Holistic Health Practice

**DOI:** 10.1100/tsw.2006.357

**Published:** 2006-12-15

**Authors:** Erica Bell

**Affiliations:** University Department of Rural Health, University of Tasmania, Australia

**Keywords:** research methodology, holistic health practice, whole-of-patient approaches, Australia

## Abstract

Holistic health practice is often described as being about understanding the larger contexts of patients, their health services, and their communities. Yet do traditional quantitative and qualitative health research methods produce the best possible evidence for the holistic practices of doctors, nurses, and allied health professionals? This paper argues “no”, and examines the potential of a cutting-edge, social science research method — Quali-Quantitative Research (QQA) — for providing better evidence for holistic practice, particularly in small-N populations, such as rural and remote communities. It does so with reference to the international literature on holistic medicine, as well as three holistic health projects conducted in Tasmania: about prevention of falls in older people, adolescent substance abuse, and interventions for children aged 0—5 exposed to domestic violence. The findings suggest that much health research fails to capture rigorously the contextual complexity of holistic health challenges: the multiple different needs of individual patients, and the interprofessional approaches needed to deliver multidisciplinary and multiservice health interventions tailored to meet those needs in particular community contexts. QQA offers a “configurational”, case-based, diversity-oriented approach to analysing data that combines qualitative and quantitative techniques to overcome the limitations of both research traditions. The author concludes that QQA could open new frontiers for holistic health by helping doctors, nurses, and allied health professionals answer a fundamental question presented by complex health challenges: “Given this set of whole-of-patient needs, what elements of which interventions in what services would work best in this particular community?”

## INTRODUCTION

Holistic health practice is often described as being about understanding the larger contexts of patients, their health services, and their communities. Such approaches have also been associated with "person-centred care": patent-sensitive health care services informed by richer understandings of patients, their bodies, circumstances, and individual needs, over time and place[1,2,3]. Complementary and alternative medicine (CAM) therapies are often described as part of a body-mind-emotions-spirit approach to healing[4]. They include acupuncture, dietary and herbal supplements, reflexology, yoga, massage, chiropractic services, Reiki and other energy therapies, aromatherapy, therapeutic touch, intravenous chelation, ozone therapy, iridology, reflexology, and homeopathy[4,5,6].

It is estimated that most people in the world rely on CAM, which is increasingly being accepted into mainstream health practices and education; over 40% of Americans have used CAM[7]. Yet, do traditional quantitative and qualitative health research methods produce the best possible evidence for the holistic practices of physicians, nurses, and allied health professionals? This paper argues "no", and examines the potential of a cutting-edge social science research method — Quali-Quantitative Research (QQA) — for providing better evidence for holistic medical, nursing, and allied health practice. It aims to provide directions for better research for holistic practice.

## METHODS

The international literature on holistic health is briefly and selectively reviewed to develop broad understandings about research needs for better holistic practice. These papers were obtained through a PUBMED search using the terms "holistic medicine" to identify 1,645 titles. The references cited in this paper were selected on the basis of their relevance to the question of what research methodologies would provide better evidence for holistic medicine.

The paper then presents, as case studies of the evidence needs of holistic practice, the findings of three Tasmanian projects on which the author has worked: prevention of falls in older people, adolescent substance abuse, and interventions for children aged 0–5 exposed to domestic violence. These case studies further identify the limitations for holistic health practice of existing research methodologies and the kinds of evidence they produce. In particular, the case studies are used to explore how traditional quantitative approaches associated with the classical experimental research model fail to provide evidence that responds to the diversity challenges of holistic practice, i.e., the need to decide what set of whole-of-patient needs would be best met by which elements of what interventions in which services, given what environmental and community contexts. The references cited (sparingly) in this discussion are based on literature reviews conducted for these three projects.

Finally, the paper explores ways of better meeting the evidence needs of holistic practice by using research techniques developed by Charles Ragin, and associated with the global research movement QQA. The techniques of QQA are explained in ways that show why it could help to develop better evidence for holistic, whole-of-patient approaches in medical and allied health practice.

For this part of the paper, a selection was made of Ragin's work as listed on his website (http://www.u.arizona.edu/~cragin/cragin/), as well as the Compass small-N website for systematic cross-case analyses and small-n studies (http://www.compasss.org).

## RESULTS

### Research Needs of Holistic Practice

First, holistic practice needs better theorisation, beginning with better theorisation of its essential nature. Better conceptualisation of holistic practice is crucial for reflective practice, for policy decision making in health, and for distinctive areas of endeavour that involve communicating the nature of holistic practice, such as health promotion and professional education[8,9]. Research methodologies must be able to deliver better definitions of holistic practice that can displace narrow, biomedical, disease-centred definitions of health. Such definitions should offer expanded conceptual models for health that are capable of including, for example, non-Western health concepts based on mind-body integration[10]. Cultural constructions of health are changing, and broadening, such that research must reflect more complex, holistic, multidimensional understandings simply to keep pace with social expectations. We live in times where people are becoming increasingly conscious of the interacting roles of biology, mind, behaviour, environment, social situation, culture, spirituality, etc.[11].

Research should also be able to offer new insights into existing conceptualisations of holistic medicine; the meaning of consciousness-based medicine[12] and "person-centred care", which some have argued is poorly theorised in the literature[13]. Research methodologies will need to accommodate the fact that having CAM is not the same as having holistic care; evaluation of holistic approaches requires capturing the underlying philosophy of care that transforms the health care contexts, such as underpinning beliefs in patient empowerment[14]. Theorisation of holistic practice should help to ensure that holistic medicine is no longer identified in relation to traditional biomedical medicine, but rather as a form of integrative practice with its own distinctive concepts and terminology[15].

The second area of need is for better evidence to support whole-of-patient approaches. Whatever else it is about, holistic health and medicine is about whole-of-patient needs. Research methodologies for capturing evidence for holistic practice must therefore move beyond narrow biomedical models of measurement, to take into account the patient's broader situation and provide information that can help practitioners, especially those with traditional biomedical training, to appreciate the complex interactions between personal circumstances, health behaviours, and health[1]. Such information is important to "person-centred diagnosis"[16], based on understandings of the patient's physiological, environmental, and psychosocial needs, and specific understandings of antecedents, triggers, and mediators that underlie the patient's condition[16]. The evidence needed must be useable in holistic practices that focus on more complex, underlying causes in patients with multiple related conditions, rather than providing information that supports atomising biomedical approaches that consider only one condition at a time[17,18]. That is, holistic practice needs research that can support its focus on multiple conditions in the one patient, including chronic conditions that may be related to lifestyle, and the combinations of elements from different conventional and CAM approaches that are needed to make a difference to these patients[19,20]. This is only saying that research must reflect what practitioners are saying, which is that biomedical approaches by themselves often do not work to restore the health, well-being, and quality of life of patients with chronic conditions[21]. Accordingly, there is a need for research evidence that helps the practitioner to better respond to the whole story of the patient's condition, including its complex history[11,16,21].

A third area of need is for better research evidence for particular areas of holistic practice for particular groups. While research methodologies for CAM must reflect a focus on the individual's needs and characteristics, not population health characteristics[22], they should also be capable of examining areas of practice that develop in response to conditions shared by groups of people. For example, there is a need for better information about the nature and role of "consciousness-based medicine"[12] and how its focus on personal development works for specific groups, such as children[23] and adolescents[24], when biomedicine is unhelpful or counterproductive. The efficacy of CAM for older people with chronic conditions remains substantially under-researched, although there are indications that chronic conditions and disability are reliable predictors of CAM use[25].

A fourth area of need in holistic health and medicine is for rigorous evidence for specific CAM therapies, including their interactions with mainstream medicine, to support integration of these approaches into health care environments[5]. As more people turn to CAM, explanatory models that offer insights into the mechanisms of action and interaction involved in their effectiveness are highly desirable[21,26].

Such research for holistic practice must engage with the idea that a health intervention is not about specific therapeutic packages; it is about the whole client's experience of the health service and healing. Accordingly, research for holistic practice is likely to open up new areas for enquiry; for example, in relation to the psychosocial aspects of healing and their relationships with other interventions[27], or in relation to healing landscapes and the relationships between these spaces and health[3,28,29]. Being able to capture the whole patient's experience means being able to accommodate the different time frames needed to observe the effectiveness of holistic approaches[30].

The search for better evidence for specific CAM therapies and their interactions should not lead to atomisation of the contribution of each CAM or mainstream therapy. New methodologies are needed that account for the view in holistic medicine that an integrated whole is more than the sum of its parts, which interact synergistically to achieve the observable effects[11]. Across quite diverse health challenges from chronic conditions, such as diabetes, to diagnostic health uncertainty, to health promotion, more complex nonlinear approaches to understanding causality are needed that do not rely on breaking down complex systems into constituent parts. Research evidence is needed to support clinical decision making and problem solving that engage with this complexity and synergy[31], and to inform, for example, individualised treatment plans that reflect this holistic mindset[16].

Holistic practice is also often highly combinatorial. Accordingly, research into CAM therapies needs to reflect the fact that the holistic practitioner may combine elements of different CAM and mainstream therapies in different ways to meet individual needs. That is, the holistic practitioner is ideally a responsive practitioner with a wide range of healing repertoires that reflect the emphasis on an integrative "body-mind-emotions-spirit" approach[12]. Today, good clinical practice, as much as holistic practice, is multiparadigmatic, using different combinations of tools from different toolboxes, such as classical, manual medicine, biomedicine, and holistic or consciousness-oriented medicine[30].

If research on CAM therapies needs to reflect the whole patient's experience, and the complexity, synergy, and combinatorial nature of holistic practice, such research also needs to engage with the challenge that some aspects of CAM have indefinable qualities. An example might be spiritual therapies, which are seen by many holistic practitioners as an important part of CAM[14,32]. Spiritual therapies have an important role to play in positive health outcomes[32]. Undoubtedly, rigorous approaches are needed that can test arguments that CAM approaches have not been demonstrated to have any "scientific value"[32]. At the same time, research methodologies need to be sufficiently "open-ended" to account for the fact that not all aspects of health and healing can be explained[11]. Western rationalist scientific thought is most commonly predicated on the idea that everything can be subjected to analysis and measurement. Many aspects of holistic practice can and should be explained in the interests of more informed practice, but at the same time, a rigorous model of evaluation need not necessarily be a closed model of thinking and measurement.

Finally, the evidence for holistic practice must have authenticity or "external validity" (not simply internal validity), particularly for service delivery contexts. This is part of the challenge of capturing diversity. In particular, research for holistic practice needs to reflect and inform holistic models of interprofessional services[1]. In holistic health service contexts, the patient will typically see a number of different professionals, including body therapists, psychotherapists, and physicians trained in using holistic approaches[12]. Interprofessional models of health care characterise rural contexts[33]; developing better evidence for them will help better serve these regions.

The three case studies that follow share learnings from Tasmania, Australia. They offer further exploration of the research needs of holistic practice, building on the previous discussion. Tasmania is a temperate island approximately the size of the republic of Ireland, around 250 km off southeast Australia, with a relatively decentralised population of around 485,000. Much of the island presents as a pastoral idyll, with historic convict-built towns and small farms with sheep grazing. The island has the highest proportion of environmentally conscious or "green" voters of all Australian states, and 40% of the island is held in parks and world heritage areas. The projects that follow took a particular focus on the needs of rural communities. Their holistic approach suggests a feature of university departments of rural health in Australia — our engagement with multidisciplinary health challenges in our local communities.

## Case Study 1: Falls Prevention

The project aimed to support improved falls prevention in family practices in Tasmania through education and training. Falls prevention is recognised as a complex health challenge for which holistic approaches are needed, engaging with its physical, psychological, and environmental aspects in older patients presenting with multiple chronic conditions and multifactoral risk profiles[34,35,36,37,38,39,40,41]. Yet such evidence to support holistic practices has not been so well translated into clinical practices[42].

Several training and education approaches were implemented that aimed to support more holistic, multidisciplinary approaches to falls prevention. These included development of a clinically focussed education package for state-wide use, clinical demonstration clinics, and the support of ten family practices in regional and rural parts of Tasmania to implement the Quickscreen©Clinical Falls Risk Assessment Tool ("the

Quickscreen tool") developed by the Prince of Wales Medical Research Institute (http://www.powmri.edu.au/contact.htm). The Quickscreen tool is a tool for assessing falls risk in clinical contexts. It allows practitioners to calculate falls risk from factors such as previous falls, medication use, vision, peripheral sensation, lower-limb strength, balance, and coordination. Standardised semi-structured questionnaires and interviews were conducted with practitioners participating in these education and training initiatives to obtain evaluation information[43]. The project offers a number of key learnings for future research into holistic practices. It suggested that holistic falls prevention needs better theorisation, including about its essential nature, in ways that can be translated into family practice contexts. This theorisation should provide practitioners with concepts and terminology for integrative practices that address the physical, psychological, environmental, and community-based aspects of falls. While the Quickscreen tool was useful in helping nurses in this study respond to the whole patient, there was no evidence that it helped developed multidisciplinary, collaborative, case-management teams involving other professionals, including physicians[43]. Other kinds of learning opportunities and tools are needed for this to happen, that facilitate better understandings of what is holistic, interprofessional practice and why it is best practice. Practitioners in falls prevention also need systematic evidence about the different combinations of interventions, covering the full range of CAM for holistic practice[44]. The Quickscreen tool identifies individuals at risk of falling, but more and better information is needed about how these risk factors interact in synergistic ways as part of complex causality in falls. Such information should be in a form that reflects the nature of decision making in holistic falls prevention.

## Case Study 2: Designing a Residential Service for Adolescents with Substance Abuse Issues

The aim of this project was to develop a residential service blueprint for adolescents with substance abuse issues in Tasmania. Tasmania does not have a residential service for adolescents with substance abuse issues, although most other Australian states do. The project developed a package of advice, including service design blueprints, for an organisation wanting to develop a best practice state-wide residential service for adolescents (12–18 years) in Tasmania. The researcher was contracted by a nongovernment organisation to specify the features of an ideal, but practicable, service — its service mission, programs, and services to be offered; ideal location and architecture; staff profile; and community benefits.

The researcher undertook an extensive literature review and consultation process: interviews and focus group meetings with 60 youth services professionals (including health practitioners), as well as 39 youth, in Tasmania. Interviews were also conducted with 21 staff in 20 of Australia's residential services for adolescents with drug issues. The consultations involved the use of open-ended questions that invited study participants to reflect on their ideal service. This study also offered key learnings for future research into holistic practices, in a context where all study participants emphasised the value of a holistic service. Youth indicated that it was important to understand the duplicity of global and social forces that simultaneously tempt and prohibit drug use. They advised against simplistic monocausal theories of drug use based on, for example, family agency[45]. Adult practitioners spoke about the importance of combining diverse elements from mainstream medicine as well as CAM approaches[46]. For research into holistic health practice, this project indicates the great gap between classical experimental studies based on large quantitative datasets and the needs of practitioners for configurational evidence that helps to answer the question: "What elements of which services and mainstream or CAM therapies, would work well for this patient with this particular set of needs, in this context?" That is, this study emphasised the importance of research that can capture the diverse, combinatorial nature of good practice in residential service delivery.

## Case Study 3: Developing Services for Children (0–5 Years) Exposed to Domestic Violence

This project aimed to develop a best-practice framework for responding to the needs of children 0–5 years old that were exposed to domestic violence, presenting to emergency accommodation services for women in Tasmania. Domestic violence is a complex social problem that involves a range of behaviours, as well as their consequences for (mostly, but not exclusively) women and their children[47,48,49]. There is growing evidence that small children, including unborn children, experience particular consequences from their exposure to this violence in its many forms, especially when combined with other risk factors, such as mental health problems, lower levels of social competence, behavioural problems, poorer academic and verbal functioning, diminished neurocognitive functioning, as well as long-term negative outcomes[50,51,52,53,54,55,56,57]. Such research supports the value of early intervention. The emergency accommodation services involved in this study delivered a broad range of services that included crisis assessment and support, information and referral, emergency accommodation, and long-term and transitional support. The Tasmanian government has recognised, in its policy in this area, the difficulties that services have had responding collaboratively to the holistic needs of small children exposed to domestic violence. The study was designed to help move practices forward in Tasmania in this area, by working collaboratively with service staff.

Five of Tasmania’s six emergency accommodation services for women were involved in the study. An extensive international literature review was conducted, identifying best practice in key areas: service ethos and mission; assessment; multiservice collaboration; as well as interventions, including key children’s activities and play modules. For each of these areas, service practitioners were invited to provide an account of existing approaches in their services. They were then asked to reflect on the differences between this account and best practice identified by the literature review, and provide key directions for improvement of their services. The key learnings for future research into holistic practices from this project emerged as it became clear how difficult it is to achieve services development without a body of evidence supporting that development. The domestic violence research internationally takes a very diagnostic approach, and is focussed on such questions as “What is domestic violence?” “What are its effects?” There is not enough nuanced information about specific interventions for particular clients in particular service delivery contexts. Service practitioners want and need to combine diverse service elements and work in collaborative teams, including with medical and allied health practitioners, but they lack a body of evidence to support and extend holistic approaches. More classical experimental studies (randomised control treatment studies or RCTs) of particular interventions would not necessarily meet these needs, however notable their absence is for interventions for this age group. What is needed is service-oriented research that supports the efforts of different health and social services to work together, by modelling different kinds of collaborative approaches.

## Limitations of Existing Research Methodologies

The literature review and the Tasmanian case studies suggested the need for research methodologies that can help to meet particular needs — for better theorisation of holistic practice; for individualised “whole-of-patient” approaches, but also for particular areas of practice for groups; for better evidence for specific CAM therapies that captures the whole patient’s experience, as well as the complex causality, synergy, and combinatorial nature of holistic interventions, in ways that have authenticity for service contexts. In a sentence, what is needed is evidence that is responsive to the diversity of holistic practice. What research approaches might meet these needs for diversity-oriented evidence?

Medical research remains very much dominated by biomedical approaches to health that exclude wider psychological and social dimensions, though it has been criticised since the 1970s at least for its “somatic reductionism”[58]. Traditional quantitative techniques used in classical experimental studies suffer from methodological errors that limit not only their usefulness for holistic practice, but for what is generally accepted as good modern clinical practice engaging with the demands of complex health conditions; an

"exaggerated search for certainty", atomisation and dehumanisation of the researcher (and the research subject), deterministic linearity, reductionism and atomisation of the whole, errors of induction, "exaggerated mathematisation", narrow and inflexible "criteria for truth", errors to do with "restricting the sources of truth and ways of searching for truth", discarding of odd or awkward facts[59]. For example, errors of induction and narrow criteria for truth characterise much research relying on statistical tests of probability routinely performed in "point and click" quantitative recipe approaches. Questions have rightly been asked about such approaches, such as "Why do we search for the truth with a probability of 95%? Can an inductive conclusion with a probability amounting to 50 or 75% be truthful?"[59]. The nursing literature includes substantial discussion of how the "truth value of evidence" is constructed by existing disciplinary knowledge, and the limitations of reductive scientific methodologies for delivering the multidisciplinary evidence required by holistic, patient-centered nursing practice[60]. In medicine, there are calls to put aside the scientific paradigm that has been with us since Newton, driving RCTs, and develop a new "nonlinear, holistic, complex way of thinking" in medical research, to accommodate the fact that biomedical phenomena are themselves complex, dynamic, and nonlinear[59,61]. There are critiques of RCTs and the quantitative research techniques associated with them, which suggest these techniques are too reductive to accommodate the paradigm shift that is happening in medical practice, and the complexities of good modern clinical practice, including for chronic diseases[17,18,61,62]. It seems unlikely that traditional quantitative methodologies associated with RCTs will be abandoned, yet the idea is spreading that they should be considerably transformed if they are to serve modern health and medical practice properly.

Qualitative approaches typically used in holistic nursing research, such as grounded theory, content analysis, and narrative analysis[63], bring a richness to understandings of human experience. However, they remain vulnerable to dismissal on the grounds that they lack generalisability and reliability. By themselves, traditional qualitative approaches also seem unlikely to deliver the rigorous evidence base needed for holistic practice.

If holistic approaches using CAM are to be integrated into mainstream medicine, or even if some of them continue to stand outside that mainstream, they need more and better styles of evidence beyond what traditional qualitative and quantitative research methodologies can provide, driven by new developments in research methodology[15]. For this reason, this paper will now consider one of the more promising methodologies to emerge from the social sciences — Quali-Quantitative Analysis.

## Quali-Quantitative Analysis

This section describes the main features of QQA and what a holistic study using this method would look like. QQA is a systematic approach to analysing cases considered as configurations. It is a set of techniques that allows rigorous, but also holistic, comparisons of cases to show their similarities and differences. It is of interest to holistic health and other fields because, as a transdisciplinary method, it aims to combine elements of both quantitative and qualitative techniques to overcome the limitations of both traditions (hence the use of the term "quali-quantitative analysis" in this paper, instead of Ragin's term "qualitative comparative analysis"). As this paper has suggested, the limitations of both quantitative and qualitative traditions have now created something of a "fork in the road" in health research.

QQA is a global research movement that began with Charles Ragin's much lauded 1987 book *The Comparative Method*[64]. The approach has been used in over 250 applications across different disciplines, but its value for health research has only just begun to be explored[65]. The fact that health remains one of the few areas impervious to QQA perhaps suggests something about the methodological conservatism of health research cultures deeply persuaded of the value of traditional quantitative techniques in classical experimental studies. Ragin is concerned with how to handle small datasets to produce rich cross-case evidence that captures complex causality. His whole enterprise really casts doubts on any attempts to obscure this complexity in the kind of homogenising, variable-driven research that too often characterises classical experimental studies[66].

The techniques for a QQA approach are given in diverse writings by Ragin and his colleagues[64,66,67,68,69,70,71]. However, readers are directed to the fullest and latest account of his thinking given in his 2000 book *Fuzzy-Sset Social Science*[66], as well as to the software developed for these analyses available at Ragin's website (http://www.u.arizona.edu/~cragin/cragin/), and supporting material listed there[72]. The simplification of the method that follows is based on consideration of these references, particularly *Fuzzy-Set Social Science*[66].

There would be three basic steps in a holistic health study using QQA. The first step would be selection of cases and tabulating these cases according to similarities and differences in the characteristics that are of interest. As QQA is a method of analysis, it can accommodate a variety of data collection and sampling methods, as long as these yield the rich information required by the analysis and are defensible. That is, both qualitative and quantitative data can be collected, and cases can be selected using different sampling techniques, including for randomised control and treatment groups. Where the case characteristics of interest are "black and white", they can be tabulated in terms of their presence or absence. In many holistic health studies though, the researcher will want to capture degrees of membership of particular characteristics. Such characteristics will typically be causal conditions and outcomes observed for a particular intervention or interventions involving particular patients. The researcher will be interested in "whole-of-patient" characteristics, as well as different elements of conventional and CAM therapies, along with environmental and community characteristics. As has been suggested, in a holistic study, environmental characteristics might be about features of healing places. The QQA approach encourages iterative, collaborative approaches to research; a holistic health researcher might involve the patient in developing the characteristics for the tabulation, as well as the decisions tabulated about that patient's conditions and outcomes. Having tabulated the cases (using software), the holistic researcher would then "minimise" the cases to shorthand expressions or "logical equations" of the different kinds of combinations found. Different combinations of conditions could well produce the same outcome, and the number of logical equations produced would reflect this.

The second step in a holistic QQA study would involve tests of the "necessity" and "sufficiency" of possible causal conditions. This brings rigour and systemacy to the evidence about causality. A cause is necessary if all instances of the outcome occur with the cause, and a cause is sufficient if all instances of the cause occur with the outcome. "Probabilistic criteria" are applied at this stage, underpinned by Bayesian probability theory. However, the emphasis is on arriving at judgments that have been tested by looking at the data and considering what makes sense rather than "pointing and clicking" to follow quantitative recipes. Again, this stage can be done consensually; in a holistic QQA study, different practitioners might be involved in reviewing the researcher's judgments.

The third and final step in a holistic QQA study would be to evaluate the results of the tests of necessity and sufficiency of causal conditions. This must be done in ways that question any tendencies of simplification. The aim of this stage is to challenge reductive thinking and to seek out opportunities for falsification of the findings (about which conditions are necessary and sufficient for which outcomes). For example, in a holistic QQA study of the role of Reiki, massage, and spiritual therapies in adolescent substance abuse interventions, at this stage the researcher would examine cases where positive life changes had not been observed ("negative cases"), derive the configurations of conditions found in those cases, and compare these with the findings obtained from the primary study.

## DISCUSSIONS AND CONCLUSIONS

The challenges surrounding the need for better evidence for CAM, and for holistic practice generally, will not be resolved anytime soon, but that should not prevent researchers from energetically pursuing better methodologies. We need a culture in health research that questions the methodological status quo because traditional biomedical research approaches seem unlikely to meet the evidence needs of good practice for responding to the complex health challenges we face in the new millennium. Accordingly, researchers who want to support good practice need to be open to new methodologies from other disciplines. QQA has strengths and limitations that really need to be explored in different kinds of health studies, but its application in so many other disciplines now challenges health researchers to explore its usefulness. It offers a configurational, diversity-oriented approach to analysing data that combines qualitative and quantitative techniques to potentially overcome the limitations of both research traditions. Those limitations seem to be holding back the evidence base for holistic medicine. The great strength of QQA for holistic health practice appears to lie in its ability to provide rigorous configurational evidence about interactions between patients, health services, and their interventions, and wider community and environmental contexts, particularly in small-N populations. For this reason, it should be of particular interest to those who want to see better evidence from, and for, rural and remote health contexts.

QQA could help to open new frontiers for holistic health by creating a stronger evidence base for holistic responses to a whole range of complex 21$^{st}$-century health challenges. It promises to provide better answers to the pragmatic question asked by doctors, nurses, and allied health professionals when faced with holistic health challenges: “Given this set of whole-of-patient needs, what elements of which interventions in what services would work best in this environment and community?”

## ACKNOWLEDGEMENTS

In relation to Case Study 1, the author (who was the evaluator for phase two of the project) would like to acknowledge the roles of Maree Fish (who was the principal project officer) and Christine Stirling (who was the evaluator for phase one). Thanks are also due to the study participants, reference group, and project partners. The project, entitled Stand Up Right – Stay Upright! – Building falls prevention capacity through workforce development, formed part of a national Australian falls prevention project for older people funded by the Australian Government Department of Health and Ageing.

In relation to Case Study 2, the author was sole researcher. Acknowledgements are owed to the project reference group, particularly Nell Kuilenburg in her role as Director of Accommodation and Support Services for The Salvation Army (Tasmania). The project was commissioned by The Salvation Army (Tasmania) and funded by the Commonwealth Department of Health and Ageing’s Illicit Drug Diversion Initiative, and The Tasmanian Department of Health and Human Services.

In relation to Case Study 3, the author was sole researcher. Acknowledgements are owed to the staff of emergency services for women in Tasmania who participated in the study. The study was commissioned by The Salvation Army (Tasmania) and funded by the Supported Accommodation Assistance Program jointly funded by the State and Commonwealth Governments.
